# Caffeine Improves Basketball Performance in Experienced Basketball Players

**DOI:** 10.3390/nu9091033

**Published:** 2017-09-19

**Authors:** Carlos Puente, Javier Abián-Vicén, Juan José Salinero, Beatriz Lara, Francisco Areces, Juan Del Coso

**Affiliations:** 1 Exercise Physiology Laboratory, Camilo José Cela University, 28692 Madrid, Spain; carlos.puente@sek.es (C.P.); Javier.abian@uclm.es (J.A.-V.); jjsalinero@ucjc.edu (J.J.S.); blara@ucjc.edu (B.L.); fareces@ucjc.edu (F.A.); 2 Performance and Sport Rehabilitation Laboratory, University of Castilla La Mancha, 45071 Toledo, Spain

**Keywords:** ergogenic aids, stimulants, team sport, elite athlete, side effects

## Abstract

The aim of this study was to determine the effect of caffeine intake on overall basketball performance in experienced players. A double-blind, placebo-controlled, randomized experimental design was used for this investigation. In two different sessions separated by one week, 20 experienced basketball players ingested 3 mg of caffeine/kg of body mass or a placebo. After 60 min, participants performed 10 repetitions of the following sequence: Abalakov jump, Change-of-Direction and Acceleration Test (CODAT) and two free throws. Later, heart rate, body impacts and game statistics were recorded during a 20-min simulated basketball game. In comparison to the placebo, the ingestion of caffeine increased mean jump height (37.3 ± 6.8 vs. 38.2 ± 7.4 cm; *p* = 0.012), but did not change mean time in the CODAT test or accuracy in free throws. During the simulated game, caffeine increased the number of body impacts (396 ± 43 vs. 410 ± 41 impacts/min; *p* < 0.001) without modifying mean or peak heart rate. Caffeine also increased the performance index rating (7.2 ± 8.6 vs. 10.6 ± 7.1; *p* = 0.037) during the game. Nevertheless, players showed a higher prevalence of insomnia (19.0 vs. 54.4%; *p* = 0.041) after the game. Three mg of caffeine per kg of body mass could be an effective ergogenic substance to increase physical performance and overall success in experienced basketball players.

## 1. Introduction

Elite athletes typically use different nutritional and ergogenic strategies to improve sport-specific performance, and caffeine is one of the most consumed substances because of its effectiveness to increase physical performance in both team and individual sports [1,2,3]. In fact, data obtained from urine samples used to detect doping have revealed that three out of four elite athletes use this substance before or during competition [4]. The high use of caffeine in sports is related to both social and physiological factors: Caffeine and caffeinated products are commercially available at a very low price, its consumption is frequently associated to social behaviors and the world of sport has always had a high tolerance for the use of this stimulant [5]; in addition, caffeine is rapidly absorbed with peak plasma concentration 40–80 min after its ingestion, while caffeine can interact with many tissues in the body, producing a myriad of physiological effects in several organs (skeletal muscle, liver, heart, and adipose tissue) [6]. In sports, the stimulant effect of caffeine in the central nervous stimulant, acting through the blockade of central and peripheral adenosine receptors [7], is considered as the most common mechanism of action related to increased physical and sports performance, although it is not the only mechanism proposed [6,8]. All of these physiological outcomes suggest that caffeine’s stimulant effect is rapidly perceived by athletes, while this substance possesses the potential capacity of affecting performance in a wide variety of sport disciplines [9].

Perhaps one of the most sought-after effects of caffeine intake in team-sports athletes is the enhancement of high-intensity movements and running speed during sprints, because of the relationship of such actions with overall success in these types of sports [10,11,12]. To date, inconsistent effects of caffeine have been obtained during sprint tests that included several repetitions at maximal running speed, in an attempt to replicate the conditions of team-sports competition [13,14,15,16]. However, caffeine intake has repeatedly been found effective to increase the number of movements and distance covered at high-intensity during simulated and real rugby [10,13], soccer [11,14], hockey [17] and volleyball games [1,18].

Although the scientific information about the effects of caffeine on basketball performance is scarce, a few specific skills of this team sport have already been analyzed after the ingestion of this substance [19,20]. One of these studies showed that caffeine (3 mg/kg body mass) did not increase VO_2max_ nor jump height during a 10-vertical jumps test performed by five basketball players [20]. On the contrary, sixteen elite young basketball players improved their jump height in single and repeated maximal countermovement jumps after the ingestion of the same dose of caffeine [19]. Because jump performance is a key variable for basketball success, the increased jumping ability after caffeine intake might enhance overall basketball performance, but this has not been investigated yet.

Apart from excellent abilities to repeat sprints and jumps, other attributes are fundamental for experienced players to be successful in a complex sport such as basketball. Among them, cognitive and skill-based demands, along with decision-making components, are critical for basketball-specific actions, such as dribbling, shooting and passing. While the effects of caffeine to increase physical performance are well-recognized, the potential effects of caffeine on accuracy and decision-making actions in skill-based sports is unclear [21,22,23,24,25]. On the one hand, caffeine intake did not exert any influence on accuracy in clay target shooting [21], on skills and technical performance during a match in female elite rugby sevens players [22], nor passing accuracy during intermittent shuttle-running trials that simulated soccer play when ingested in combination with carbohydrate [23]. On the other hand, caffeine ingestion improved passing accuracy during a high-intensity intermittent rugby test [24] and during a simulated soccer game when this substance was ingested alone [25]. To date, only one investigation has studied the influence of caffeine intake on shooting accuracy in junior basketball players [19]. In this investigation, caffeine did not exert any influence—either positive or negative—on the accuracy of free throws and three-point shots.

With this background, it is difficult to confirm whether caffeine is an ergogenic aid for basketball players because the effects of this substance on basketball-specific physical and/or technical actions (sprinting, shooting abilities and game performance indicators, etc.) are not well established. The aim of this investigation was to determine the effects of caffeine intake on overall basketball performance in experienced players assessed during specific tests and during a simulated basketball match. We hypothesized that the pre-exercise ingestion of 3 mg of caffeine per player’s body mass would increase physical performance in basketball-specific tests, but it would not affect game-related statistics associated to improved overall basketball performance (field goals accuracy, rebounds, assists, etc.) during a simulated basketball game.

## 2. Materials and Methods 

### 2.1. Participants

Twenty well-trained, experienced basketball players, from two different basketball teams, volunteered to participate in this investigation. The sample included 10 professional female basketball players (age: 27.9 ± 6.1 years) and 10 semiprofessional male basketball players (age: 27.1 ± 4.0 years). We selected both female and male basketball players to allow the application of the outcomes of this investigation to both sexes. All participants had prior basketball experience of at least 10 years and had trained for approximately 2 h/day, 5 days/week (including a weekly competition) during the previous year. The exclusion criteria were as follows: Previous history of cardiopulmonary diseases, taking medications or sympathetic stimulants during the experiment or having suffered a musculoskeletal injury in the 3 months prior to the competition. Players had no, and were not taking, medications during the duration of the investigation. Moreover, all participants were non-smokers and light-caffeine consumers (<100 mg/day). All female participants that took part in this investigation were tested during the luteal phase of their menstrual cycle. Before enrolling in the study, players were fully informed of any risks and discomforts associated with the trials and they gave their informed written consent to participate. The investigation was approved by the University Ethics Committee (code UCJC-2014) in accordance with the latest version of the Declaration of Helsinki.

### 2.2. Pre-Experimental Procedures

One week before the experimental trials, players underwent a physical examination to ensure that they were in good health. After that, participants were nude weighed (±50 g, Radwag, Poland) to individualize caffeine doses. On the same day, the anthropometric characteristics were registered by an anthropometrist, certified by the International Society for the Advancement of Kinanthropometry (ISAK) and following the methods proposed by this society [26]. Body fat was calculated from six skinfold measurements (triceps, subscapular, umbilicus, suprailium, thigh, and lower leg) according to the equations proposed by Carter [27]. Body muscle mass was calculated by subtracting fat mass [27], bone mass [28], and residual mass [29] from total body mass. The participants were encouraged by the investigators to abstain from caffeine ingestion in any form (coffee, cola, energy drinks) during the whole duration of the investigation. The day before each experimental trial, participants refrained from strenuous exercise and adopted a similar diet and fluid intake regimen. They were also instructed to have their habitual pre-competition meal at least three hours before the onset of the experimental trials, with proportions of 60/16/24% for carbohydrate/protein/fat (CESNID, Barcelona, Spain). All of these standardizations were reported to the technical staff of the basketball team to ensure compliance, and were confirmed with individualized dietary and training diaries.

### 2.3. Experimental Design 

A double-blind, placebo-controlled, randomized and counterbalanced experimental design was used in this investigation. Each participant took part in two trials under the same experimental conditions. One week of rest was set between experimental trials to allow complete recovery and caffeine washout. The experimental testing was carried out at the same time of day (from 7 to 9 p.m.) with the same ambient conditions (indoor facility at 18.5 ± 0.8 °C dry temperature; 30.8 ± 1.0 relative humidity). On one occasion, participants ingested 3 mg of caffeine per kg of body mass (3 mg/kg; 99% purity, BulkPowders, Colchester, UK; women: 191.3 ± 76.7 mg; men: 268.4 ± 40.6 mg) in an opaque and unidentifiable capsule. On another occasion, participants ingested an identical opaque capsule filled with a placebo substance (cellulose). The capsule was ingested 60 min before the onset of the experimental trials to allow complete caffeine absorption [30]. The order of the experimental trials—caffeine or placebo—was randomized and counterbalanced. However, the order of the experimental trials was set so that all the players from the same basketball team received the same treatment (caffeine or placebo) in order to facilitate the analysis of the game statistics related to each basketball team. The capsules were prepared by an investigator who did not take part in the experimental trials, who assigned an alphanumeric code to each trial to blind participants and researchers to the substance ingested by each team. This code was unveiled after the analysis of the variables.

### 2.4. Experimental Protocol

On the day of testing, participants arrived at their habitual basketball court 75 min before the beginning of the trial. They then ingested the capsule assigned for the trial. They wore their habitual competition clothes (T-shirt, shorts and basketball shoes). A GPS/Accelerometer/HR device inserted in an adjustable neoprene harness (GPS, SPI PRO X, GPSports^®^, Canberra, Australia) was provided to each player, and a heart rate monitor (Polar^®^ T31, Kempele, Finland) was firmly attached to their chest. Each player adjusted the harness and the heart rate band to avoid any hindering of movements. Players wore the same GPS unit for each experimental trial to reduce measurement error. Then, players performed a standardized and specific warm-up for 30 min led by their strength and conditioning coach. After the warm-up, players began the performance tests, which consisted of 10 repetitions of a combination of jumps, sprints and shooting tasks (2 min of recovery between repetitions). In each repetition, basketball players performed an Abalakov jump (Optojump Next; Microgate, Bolzano, Italy; DSD Sport System, Spain; [31]) followed by the Change-of-Direction and Acceleration Test (CODAT) [32]; and two free throws. There was no recovery time between the jump and the sprint, but a 14-s recovery period was set between the sprint and the free throws to replicate the time gap between stoppages in the game the onset of the free throw series (based on a previous analysis carried out in 100 official games in the same basketball category). The CODAT was performed without (from 1st to 5th repetitions) and with the basketball ball (from 6th to 10th repetitions) to assess the influence of caffeine on running velocity with and without the influence of controlling the ball. Verbal instructions were given to indicate the onset of each repetition, and oral feedback was given by the technical staff to encourage players to produce maximal performance in each trial. Participants were previously familiarized with the execution of this sequence of testing (Abalakov jump + CODAT + free throws) during training practices in the weeks before the experimental trials.

Twenty minutes after the basketball-specific testing, players participated in a simulated game played on an official basketball court. The game consisted of two parts of 10 min with a break of 2 min between them, following the rules of the International Basketball Federation (FIBA; except for the game duration). Two professional referees made the decisions during the games. Each basketball team was composed of the same individuals on both days: one guard, two forwards and two centers. The teams were previously prepared by the technical staff to create a tight game between the teams. Time-outs and changes among players were not allowed, and for this reason the game had a duration shorter than an official basketball game. In each game, players were instructed to use an individual defense and to attack without predetermined game systems. The simulated games were video-recorded by using two video cameras (Sony Handycam HDRXR200VE; Sony, Tokyo, Japan), set diagonally (approximately 10 m at the back of each half-court, and placed at 2 m above the floor to obtain a clear vision of the play). Afterwards, each game action was analyzed by two specialists in basketball game-related statistical notation by using a standard PC (Envy, HP, Palo Alto, CA, USA). For this analysis, the two specialists independently notated the basketball games by allocating the number of game actions performed by each player during the whole game, while they were blinded to the treatments and for the purposes of the investigation. In the case of disagreement between observers, the observers re-visualized the specific action together and discussed it until they obtained a final decision/evaluation. The intra-class correlation coefficient between the observers was 0.99. The following game-related statistics were gathered: Total points, free throws and two- and three-point field goals (made, attempted and accuracy), offensive, defensive and total number of rebounds; assists, steals, turnovers; received and committed blocks, dunks, and received and committed fouls. With all these variables, the performance index rating was calculated ((points + total rebounds + assists + steals + blocks committed + fouls received) − (missed shots + turnovers + fouls committed)) as proposed by the FIBA [33]. Moreover, body impacts and heart rate were continuously monitored during the game using GPS/Accelerometer/HR devices with indoor settings. Player-impact data were gathered from the accelerometer and measured in “G” force units. Although this measurement was unspecific to differentiate among different basketball actions (e.g., change of directions, jumps, etc.), it allowed the quantification of individual body movements during the game in each experimental trial. Impact intensity was graded according to the following scale: Zone 1 ≤ 0.99 G; Zone 2 from 1.00 to ≤1.99 G; Zone 3 from 2.00 to ≤2.99 G; Zone 4 from 3.00 to ≤3.99 G; Zone 5 from 4.00 to ≤4.99 G; Zone 6 ≥ 5.00 G.

At the end of the basketball game, players were required to fill out a questionnaire about their sensations of muscle power, endurance, and overall perceived exertion (RPE) during the game. This questionnaire included a 1- to 10-point scale to individually assess each item (1 point meant the minimal amount of that item and 10 points meant the maximal amount of the item); it has been previously used to assess the perceived ergogenicity of caffeine in different sport disciplines [9]. In addition, participants were provided with a survey to be filled out the following morning about sleep quality, nervousness, gastrointestinal problems, and other discomforts perceived during the hours after the game. This survey included seven items on a yes/no scale and has been previously used to assess side effects derived from caffeine ingestion in the hours following an official or simulated competition [9]. This survey also included specific questions to evaluate the success of the blinding procedure.

### 2.5. Statistical Analysis

Male and female basketball players were treated as a single group in the statistical analysis because no sex interactions had previously been found in the use of caffeine with exercise [9,34]. Data analysis was performed using the SPSS v 20.0 software (SPSS Inc., Chicago, IL, USA). First, the Shapiro–Wilk test was used to test the normality of each variable (*p* > 0.05). After that, Student’s *t*-test for dependent variables was used to establish the differences in the variables normally distributed between the caffeine and placebo. A two-way analysis of variance was used to determine differences between treatments for the Abalakov jumps, the CODAT test (treatment × repetition) and for the different accelerometry zones (treatment × zone). The McNemar test was used to detect differences in the frequencies of side effects reported after the ingestion of each treatment. The magnitude of Cohen’s effect size was calculated and interpreted using the following scale: Trivial (0–0.19), small (0.20–0.49), medium (0.50–0.79) and large (0.80 and greater). The results are presented as mean ± standard deviation, and the significance level was set at *p* < 0.05. The 95% confidence interval for the mean difference (95% CI) between placebo and caffeine was also calculated.

## 3. Results

The anthropometric characteristics of female basketball players were as follows: Height = 175.2 ± 0.1 cm, body mass = 70.9 ± 13.0 kg, body fat mass percentage = 16.8 ± 5.4% and body muscle mass percentage = 47.1 ± 4.3%). The anthropometric characteristics of the male basketball players were as follows: Height = 193.1 ± 8.8 cm, body mass = 89.5 ± 13.5 kg, body fat mass percentage = 11.8 ± 3.1%, body muscle mass percentage = 48.3 ± 2.4%.

**Figure 1:**
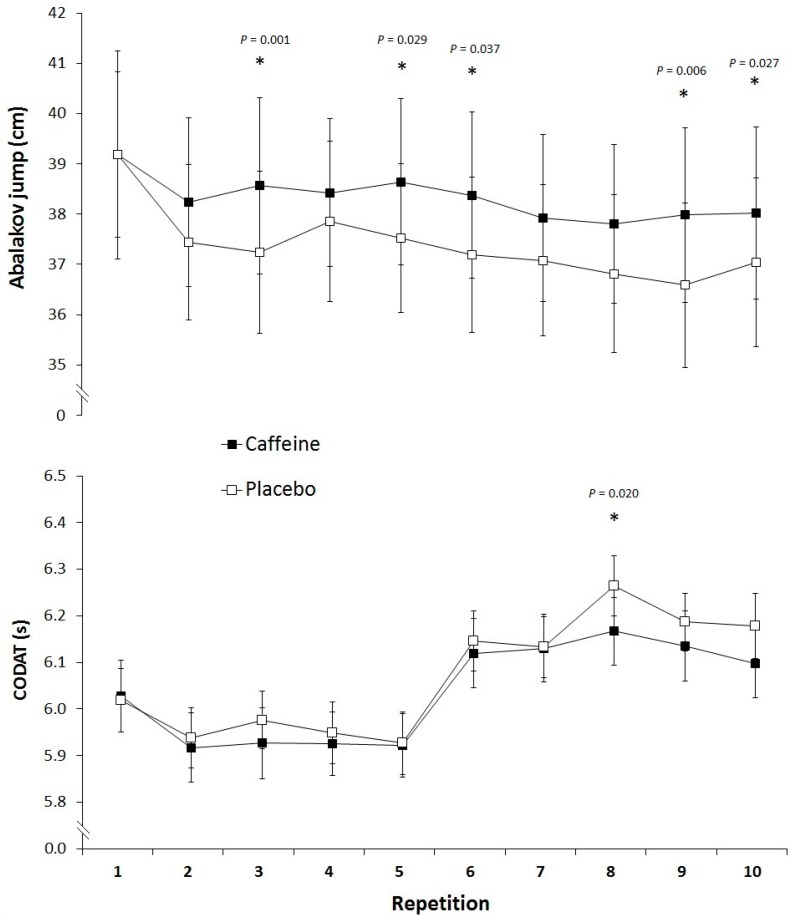
Jump height during 10 repetitions of the Abalakov jump (upper panel) and running time during 10 repetitions of the Change-of-Direction and Acceleration Test (CODAT; lower panel) with the ingestion of caffeine (3 mg of caffeine per kg of body mass) or a placebo. Data are the mean ± standard deviation for 20 basketball players. (*) Different from placebo (*p* < 0.05).

In comparison to the placebo, the ingestion of caffeine increased the mean jump height reached during the 10 repetitions of the Abalakov test (37.3 ± 6.8 vs. 38.2 ± 7.4 cm; 95% CI = 0.3 to 1.6 cm; *d* = 0.14; *p* = 0.012). Specifically, caffeine significantly increased jump height in jump numbers 3, 5, 6, 9 and 10 (all with *p* < 0.05; Figure 1, upper panel). However, there was no main effect of the repetition (*p* = 0.731) nor interaction between the treatment and the repetition (*p* = 0.561). As Figure 1 (lower panel) depicts, and even though the intake of caffeine significantly improved the 8th repetition (or 3rd repetition performed with ball; 95% CI = −0.17 to −0.02 s; *d* = 0.34; *p* = 0.020), caffeine did not change the mean time during the CODAT test without the ball (5.96 ± 0.29 vs. 5.95 ± 0.31 s; 95% CI = −0.09 to 0.07 s; *d* = 0.39; *p* = 0.388) or with the ball (6.20 ± 0.29 vs. 6.14 ± 0.32 s; 95% CI = −0.14 to 0.02 s; *d* = 0.21; *p* = 0.119). There was a main effect of the repetition in the CODAT test without the ball (*p* = 0.047), but there was no main effect of the repetition in the CODAT test with the ball (*p* = 0.481) nor interaction between the treatment and the repetition in the whole test (*p* > 0.050). With respect to the placebo, the caffeine intake did not modify the number of free throws made at the end of each repetition of the CODAT test (15.4 ± 1.6 vs. 15.6 ± 2.3; 95% CI = −0.8 to 1.1; *d* = 0.09; *p* = 0.389) and there was no effect of the repetition nor interaction treatment × repetition (*p* > 0.050).

**Figure 2:**
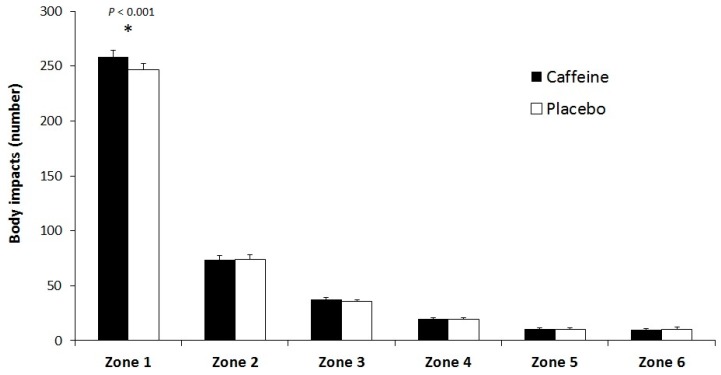
Number of impacts per minute during a simulated basketball game with the ingestion of caffeine (3 mg of caffeine per kg of body mass) or a placebo. Data are mean ± standard deviation for 20 basketball players. Zone 1 ≤ 0.99 G; Zone 2 from 1.00 to ≤1.99 G; Zone 3 from 2.00 to ≤2.99 G; Zone 4 from 3.00 to ≤3.99 G; Zone 5 from 4.00 to ≤4.99 G; Zone 6 ≥5.00 G. (*) Different from placebo (*p* < 0.05).

The notational analysis of the simulated basketball game showed that caffeine intake significantly increased the total number of impacts per minute of game play (396 ± 43 vs. 410 ± 41 impacts/min; 95% CI = 7.7 to 19.0 impacts/min; *d* = 0.31; *p* < 0.001). Figure 2 depicts the categorization of body impacts according to the intensity of each acceleration/deceleration. The ingestion of caffeine increased the number of impacts in Zone 1 (247 ± 26 vs. 259 ± 28 impacts/min; 95% CI = 7.5 to 14.9 impacts/min; *d =* 0.45; *p* < 0.001) during the game, but did not affect the remaining zones. There was a main effect for the accelerometry zone (*p* < 0.010), but there was no interaction between the treatment and the accelerometry zone (*p* = 0.313). Moreover, caffeine did not affect the mean heart rate (157 ± 13 vs. 161 ± 10 bpm; 95% CI = −6.0 to 10.9 bpm; *d* = 0.30; *p* = 0.299) or the maximal heart rate (185 ± 12 vs. 188 ± 10 bpm; 95% CI = −5.8 to 4.9 bpm; *d* = 0.30; *p* = 0.499).

Data from all game-related statistics analyzed during the simulated games are presented in Table 1. In comparison with the placebo, the pre-exercise ingestion of caffeine significantly increased the number of free throws attempted (*d =* 0.57; *p* = 0.042) and made (*d =* 0.67; *p* = 0.030), the number of offensive (*d =* 1.16; *p* = 0.020) and total rebounds (*d =* 0.64; *p* = 0.026) and the number of assists (*d =* 1.10; *p* = 0.019). The performance index rating was also significantly improved with the ingestion of caffeine (*d =* 0.38; *p* = 0.037). No significant differences were found in the remaining game-related statistics measured (*p* > 0.05).

In comparison to the placebo, caffeine intake significantly increased self-perceived muscle power (5.3 ± 1.4 vs. 6.6 ± 1.4 A.U.; 95% CI = 0.5 to 2.0 A.U.; *d* = 0.93; *p* = 0.003) during the testing. However, no significant differences were found for self-reported fatigue (5.3 ± 1.6 vs. 4.9 ± 1.5 A.U.; 95% CI = −1.1 to 0.4 A.U.; *d* = 0.25; *p* = 0.396) or the endurance perception (5.5 ± 1.2 vs. 6.3 ± 1.6 A.U.; 95% CI = −0.04 to 1.6 A.U.; *d* = 0.61; *p* = 0.058). During the 24 h following the test, players showed significantly higher prevalence of insomnia (19.0 vs. 54.4%; *p* = 0.041), while the remaining side effects (nervousness, *p* = 1.000; irritability, *p* = 1.000, activeness, *p* = 0.687; gastrointestinal discomforts, *p* = 1.000; headache, *p* = 1.000; and muscle pain, *p* = 0.687) were similar between both experimental trials.

## 4. Discussion

The main purpose of this investigation was to determine the effectiveness of caffeine (3 mg/kg) to improve overall performance in experienced basketball players. For this purpose, 20 experienced male and female basketball players volunteered to ingest caffeine or placebo pills before physical testing and a simulated game. Briefly, in comparison to the placebo, players increased their jump height during basketball-specific jumps, increased the number of body impacts and improved overall performance during the basketball game, as depicted by the performance index rating after the ingestion of caffeine. These effects were accompanied by higher perceived muscle power during testing and a tendency for a higher perceived endurance capacity. However, caffeine did not modify accuracy during two- and three-point field goals and free throws. All this information suggests that caffeine, in a dose of 3 mg per kg of body mass, might be considered as an effective ergogenic substance to increase physical and overall performance in basketball, although without any influence in shooting accuracy during the game.

Like the current study (Figure 1, upper panel), other previous investigations have found that caffeine (3 mg/kg) increases the jump height in different types of jumps in professional male badminton players [2], professional female and male volleyball players [1,18], professional female rugby sevens players [13], elite speed swimmers [35] and elite young basketball players [19]. Thus, it can be safely concluded that caffeine improves jump performance in individual and team sports-specific actions. On the contrary, caffeine did not enhance maximal running speed during the CODAT test, independently of whether this test was performed with or without the ball. Interestingly, previous investigations that have found benefits from caffeine ingestion (3–6 mg/kg) on maximal running speed during a repeated-bouts test have used protocols with linear running [14,15,36], while the CODAT test includes several changes of direction that imply continuous accelerations and decelerations during running. In addition, other investigations have also failed to find a positive effect of caffeine on other protocols that included repeated sprints in team-sports athletes [11,13,37]. Thus, to date, it cannot be concluded in objective terms whether caffeine is effective to increase sprint performance in team-sport players.

One of the novelties of the present investigation was the analysis of the influence of caffeine intake on game performance. While the effect of caffeine on physical performance has been well investigated, information about the translation of the physical benefits of this substance to overall sports performance is scarce. In this study, caffeine increased the number of free throws attempted and made, while caffeine did not improve the accuracy in this type of throw (Table 1). Besides, caffeine ingestion had no influence on two- and three-point field goals which reinforces the absence of positive/negative effect of caffeine in sport-specific accuracy [19,21] and other skill-based technical actions [22,23]. However, caffeine has been effective to increase the success of volleyball game-actions [1,18], that is likely because the increased physical performance allowed better positioning and possibilities to spike/block. In this regard, caffeine was effective to increase the number of assists and the number of total and offensive rebounds during the basketball game (Table 1). According to other investigations [38,39,40], a higher number of assists indicates better coordination, anticipation and timing in the team. Previous investigations have concluded that the number of rebounds is a key factor for basketball performance [38,39,41]. For example, the number of rebounds explained 23% of the wins during a regular season in the ACB (First division of Basket Club Association) category [38] and influenced game outcomes in a Basketball World Championship [41]. The higher number of rebounds with caffeine could be related to the improvement in jump height obtained with this substance. Moreover, caffeine significantly increased the performance index rating: a variable used by FIBA leagues and competitions to measure basketball players’ overall performance as far as game statistics is concerned [33]. In light of these outcomes, it might be possible that caffeine plays a role for enhancing teamwork and physical performance, contributing to better decision-making on court, although this hypothesis requires further investigation. Lastly, during the 20-min simulated basketball game against a team of the same level, the pre-exercise ingestion of caffeine increased the number of total impacts. All these data, merged together, indicate a clear influence of caffeine on basketball players’ movements during the game that ultimately might increase overall basketball performance, as previously found in other team sports such as volleyball [1,18].

The post-testing questionnaire about self-performance and the prevalence of adverse effects indicated that the ingestion of caffeine enhanced the perception of muscle power during testing, and tended to increase self-perceived endurance capacity. On the other hand, the pre-exercise ingestion of caffeine increased insomnia during the 24 h following its intake with no other measurable side effect. According to other investigations [11,13], the negative effect of caffeine on the sleep patterns of our players could be related to the experimental design, with all trials being performed in the evening, with less than 6 hours from caffeine ingestion to the onset of bedtime. The higher frequency of insomnia after the intake of caffeine should be taken into account when recommending caffeine to increase performance in team sports.

The pre-exercise meal timing (3 h before the onset of the experimental trials) and composition may have the potential to influence the effects of caffeine on basketball performance. It has been previously suggested that the ingestion of a pre-exercise meal may interfere with the absorption of caffeine and thus, with potential benefits derived from this stimulant on performance [42]. In the current investigation, basketball players had a high-carbohydrate meal before the trials, which constitutes a habitual pre-competition practice in elite athletes of most sport disciplines. This meal likely aided in the replenishment of muscle and liver glycogen stores, allowed adequate carbohydrate substrate for the muscle and central nervous system during the tests and simulated competition [43]. Furthermore, most studies have found that a similar dose of caffeine is ergogenic during real or simulated competition when this substance was ingested several hours after a pre-exercise meal [10,13,14,17,35,44]. All of this information indicates that caffeine can be potentially ergogenic in athletes with appropriate “carbohydrate availability” before competition. In any case, the ingestion of the meal (2–4 h before competition) should be separated from the ingestion of caffeine (45–60 min before competition) to avoid any potential interference in caffeine absorption.

The current study also presents some limitations related to the experimental design used. Firstly, the simulated basketball game was set to produce two teams with very similar physical and technical performances. However, the game was played without substitutions to obtain similar playing times among players and between experimental trials. Official basketball games allow unlimited substitutions; thus, the playing times and the physical demands in the present investigation were probably higher than during an official basketball game. To diminish this effect, the playing time was reduced to two halves of 10 min (instead of four quarters of 10 min). Secondly, all the data have been presented as a group mean: it might be inferred that caffeine is ergogenic for all basketball players. However, previous investigations have shown that some individuals do not experience enhanced physical performance after the ingestion of moderate doses of caffeine [35,42,45] which suggests that this stimulant might not exert ergogenic properties for all athletes. Thus, when using caffeine as an ergogenic aid, it is necessary to identify caffeine non-responders.

## 5. Conclusions

In summary, the pre-exercise ingestion of 3 mg/kg of caffeine improved jump height during Abalakov jumps, the amount of body impacts during a simulated game, the number of free throws made and attempted, offensive and total rebounds, and the total number of assists. All these statistics led to a higher basketball performance index rating with caffeine, which is the best indicator to assess overall basketball performance. On the contrary, this dose of caffeine also produced marginal side-effects during the following hours to the intake, mostly related to sleep disturbances. The outcomes of this investigation show that caffeine might be considered as an ergogenic substance to improve physical and overall basketball performance, since it was effective in increasing several basketball-specific skills. For the basketball training professional, the improvement of physical performance and game-related statistics during play, without negatively affecting the percentage of throws made during the game, might persuade them about the use of caffeine for high performance basketball players. However, the use of caffeine should be restricted to physical challenges—competitions and/or high-intensity trainings—to avoid habituation and dependence [46], for those players that have maximized performance through appropriate training and dietary habits, and taking into account its effects on sleep quality. The use of an appropriate dosage of caffeine and identification of caffeine non-responders is also advised to avoid the likelihood of drawbacks.

## Figures and Tables

**Table 1:** Game-related statistics with the ingestion of caffeine (3 mg of caffeine per kg of body mass) or a placebo. Data are mean ± standard deviation for 20 basketball players.

Variable	Placebo	Caffeine	Diff.	95% CI	Effect Size	*p* Value
Points	8.2 ± 6.9	8.8 ± 6.1	0.6 ± 7.0	−2.7 to 3.9	0.09	0.354
2-point field goals made	2.5 ± 2.4	2.7 ± 2.6	0.2 ± 3.2	−1.2 to 1.7	0.10	0.365
2-point field goals attempted	3.8 ± 3.0	4.5 ± 3.3	0.7 ± 4.1	−1.2 to 2.7	0.25	0.213
Accuracy in 2-point field goals (%)	54.7 ± 30.5	52.9 ± 37.2	−1.8 ± 50.2	−28.5 to 25.0	0.05	0.446
3-point field goals made	0.9 ± 1.2	0.8 ± 1.1	−0.1 ± 1.1	−0.7 to 0.4	0.19	0.273
3-point field goals attempted	2.8 ± 2.1	2.4 ± 2.3	−0.4 ± 2.1	−1.3 to 0.6	0.17	0.228
Accuracy in 3-point field goals (%)	27.4 ± 31.5	23.7 ± 27.5	−3.7 ± 33.4	−21.5 to 14.1	0.11	0.333
Free throws made	0.6 ± 0.8	1.1 ± 1.1*	0.5 ± 1.2	0.1 to 1.0	0.67	0.030
Free throws attempted	0.9 ± 1.1	1.5 ± 1.5*	0.6 ± 1.6	0.0 to 1.3	0.57	0.042
Accuracy in free throws (%)	71.4 ± 40.5	73.8 ± 20.7	2.3 ± 39.9	−34.5 to 39.3	0.18	0.440
Offensive rebounds	0.5 ± 0.6	1.2 ± 1.6*	0.7 ± 1.4	0.2 to 1.2	1.16	0.020
Defensive rebounds	2.1 ± 1.7	2.6 ± 1.8	0.5 ± 2.1	−0.5 to 1.5	0.30	0.146
Total rebounds	2.5 ± 2.0	3.7 ± 2.6*	1.2 ± 2.7	0.2 to 2.3	0.64	0.026
Assists	1.1 ± 0.9	2.1 ± 1.6*	1.0 ± 2.0	0.2 to 1.8	1.10	0.019
Steals	0.9 ± 1.1	1.2 ± 1.3	0.4 ± 1.6	−0.5 to 1.0	0.23	0.240
Turnovers	1.7 ± 1.5	1.7 ± 1.3	0.0 ± 2.1	−1.0 to 1.0	0.08	0.500
Blocks committed	0.0 ± 0.0	0.1 ± 0.3	0.1 ± 0.3	−0.1 to 0.2	0.22	0.081
Blocks received	0.1 ± 0.3	0.0 ± 0.0	−0.1 ± 0.3	−0.2 to 0.1	0.32	0.081
Fouls committed	1.3 ± 1.0	1.0 ± 0.9	0.3 ± 1.1	−0.3 to 0.8	0.22	0.165
Fouls received	1.0 ± 0.9	1.3 ± 1.0	−0.3 ± 1.3	− 0.8 to 0.4	0.20	0.253
Performance index rating	8.4 ± 8.3	11.6 ± 7.3*	3.2 ± 8.0	0.1 to 6.2	0.38	0.037

(*) Different from placebo (*p* < 0.05). Diff. = mean difference Caffeine − Placebo. 95% CI = 95% Confidence Interval for the mean difference.
